# Outcomes of vacuum-assisted closure therapy (VAC) in patients with Fournier’s gangrene: a retrospective cohort study

**DOI:** 10.1007/s11845-026-04416-w

**Published:** 2026-04-27

**Authors:** Emrah Küçük, Ahmet Furkan Akıllı, Reha Ordulu, Mesut Şengül, Muhammed Esad Kayhan, Ekrem Akdeniz, Mahmut Ulubay, Mustafa Kemal Atilla

**Affiliations:** 1 Department of Urology, Faculty of Medicine, Samsun University, Samsun, Türkiye; 2 Urology Clinic, Samsun City Hospital, Samsun, Türkiye

**Keywords:** Debridement, Fournier’s gangrene, Vacuum-assisted closure

## Abstract

**Objective:**

To evaluate the association between vacuum-assisted closure therapy and clinical outcomes, particularly mortality, in patients surgically treated for Fournier’s gangrene.

**Methods:**

This retrospective cohort study included 268 patients treated for Fournier’s gangrene between 2015 and 2025. Patients were divided into two groups: debridement alone (Group 1, *n* = 160) and debridement plus vacuum-assisted closure therapy (Group 2, *n* = 108). Demographic characteristics, comorbidities, laboratory findings, Fournier Gangrene Severity Index, intensive care unit admission, additional surgical procedures, length of stay, and mortality were compared. Multivariate logistic regression was performed to identify independent predictors of mortality.

**Results:**

Observed mortality was lower in the vacuum-assisted closure therapy group than in the debridement-only group (7.4% vs. 16.9%, *p* = 0.038). However, multivariate analysis demonstrated that only intensive care unit admission (OR 6.40, 95% CI 1.52–26.89, *p* = 0.011) and Fournier Gangrene Severity Index score (OR 5.57, 95% CI 2.91–10.71, *p* < 0.001) were independently associated with mortality. Vacuum-assisted closure therapy was not an independent predictor (OR 0.58, 95% CI 0.16–2.10, *p* = 0.409). The vacuum-assisted closure therapy group underwent more debridement procedures and had a longer hospital stay.

**Conclusion:**

Although vacuum-assisted closure therapy was associated with lower unadjusted mortality, survival was primarily determined by disease severity and intensive care unit admission. Vacuum-assisted closure therapy may improve wound management; however, its impact on survival appears influenced by baseline clinical status. Prospective studies are needed to clarify its role in Fournier’s gangrene management.

**Supplementary Information:**

The online version contains supplementary material available at https://doi.org/10.1007/s11845-026-04416-w.

## Introduction

Fournier’s gangrene (FG) is an uncommon but potentially lethal condition, characterised by the sudden onset and rapid progression of necrotising fasciitis, affecting the perineum, genital region, and perianal area. It necessitates urgent surgical intervention. The prevalence of the condition is observed to be highest in patients with predisposing factors, including but not limited to diabetes mellitus, chronic renal failure, malignancy, alcoholism, chronic steroid use, low socioeconomic status, and immunodeficiency. It is important to note that the condition continues to have high mortality rates even today [1, 2]. Early diagnosis, emergency surgical debridement, and broad-spectrum antibiotic treatment are critical in improving patient survival [3].

A significant challenge arises in the context of FG treatment, given substantial tissue loss following surgical debridement. The closure of such defects necessitates prolonged wound care, which in turn prolongs the healing process and increases the risk of additional complications [4]. At this point, vacuum-assisted closure (VAC) systems, which represent a contemporary wound treatment modality, are being utilised with increasing frequency. VAC therapy contributes to wound healing through several mechanisms, including the reduction of oedema via negative pressure, stimulation of granulation tissue formation, reduction of bacterial load, and control of wound exudate [5, 6].

Recent studies have demonstrated that the utilisation of VAC accelerates wound healing in patients with FG, reduces the preparation time for reconstructive surgery, and decreases morbidity [7–9]. However, the extant literature about the effect of VAC on mortality is quite limited, and existing studies are generally based on small patient series. Consequently, data obtained from larger series are critical in demonstrating the effectiveness of the treatment [10]. Our institution manages a substantial number of patients with Fournier’s gangrene. The objective of this study was to conduct a retrospective evaluation of treatment outcomes, the wound-healing process, and complications associated with VAC application for FG.

## Methods

This retrospective cohort study included 268 patients who underwent surgical treatment for Fournier’s gangrene between 2015 and 2025. Ethical approval was obtained from an institutional non-interventional clinical research ethics committee. The patients were categorized into two groups based on treatment modality: Group 1 included individuals who underwent surgical debridement alone, whereas Group 2 included those who underwent surgical debridement in conjunction with VAC therapy. The two groups were then compared in terms of demographic characteristics (age, gender), predisposing factors (diabetes mellitus, hypertension), laboratory parameters, Fournier Gangrene Severity Index (FGSI), intensive care unit (ICU) admission (yes/no), hospital stay duration, development of septic shock, additional surgical procedures performed (orchiectomy, colostomy), and mortality rates.

**Fig. 1:**
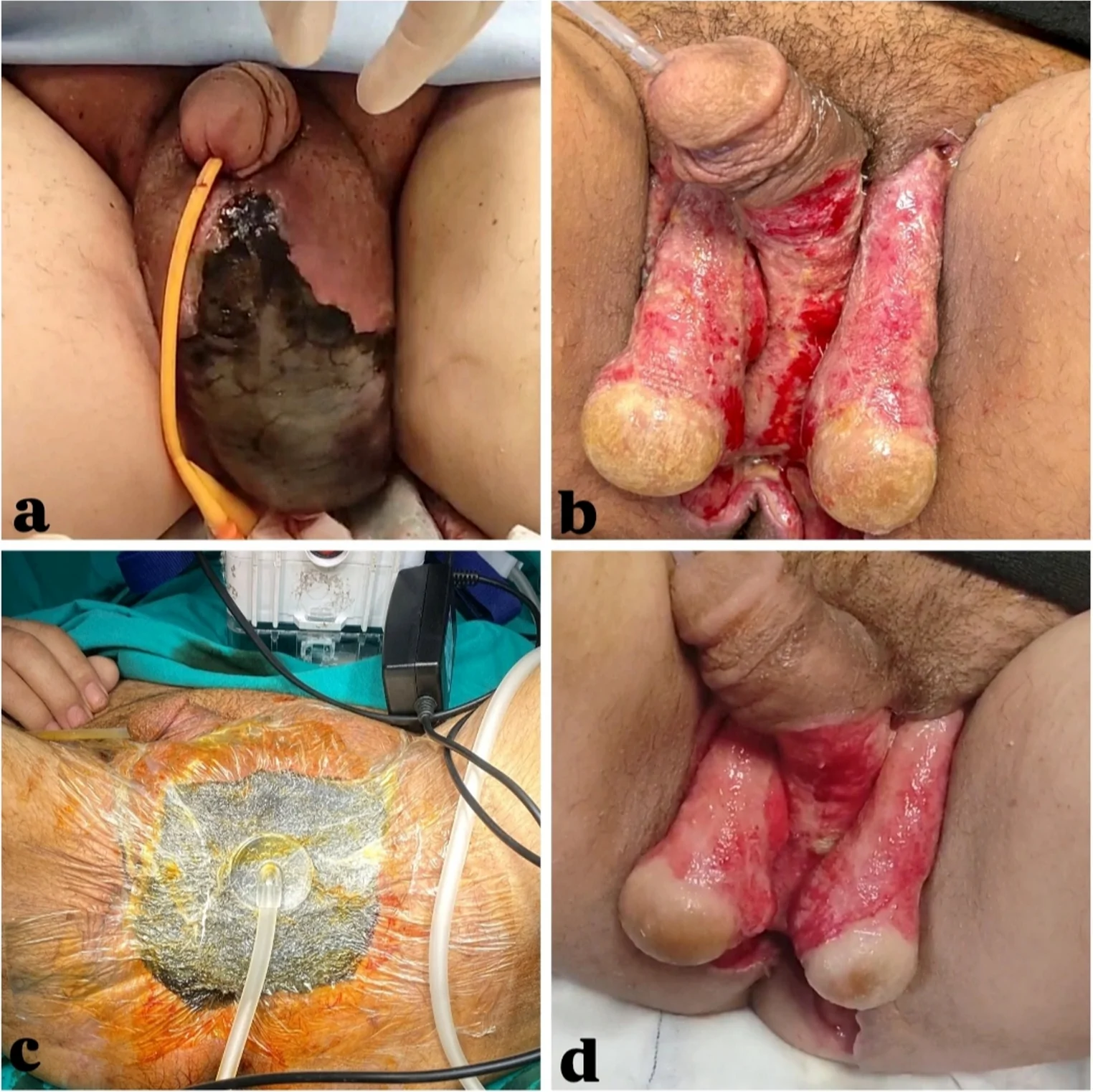
**a** Necrosis appearance in the perineal/perianal region in Fournier’s gangrene. **b** Wound area following emergency surgical debridement. **c** Wound area with VAC applied. **d ** Appearance of the wound site after VAC change

The study population comprised patients aged 18–90 years with a diagnosis of FG. Cases that had undergone major urogenital surgery for reasons other than FG or for which complete data could not be obtained were excluded from the study. The diagnosis of FG was made in all patients based on clinical examination findings (perineal/perianal tenderness, erythema, oedema, necrosis, crepitus) and laboratory results (see Fig. 1-a). Following confirmation of the FG diagnosis, emergency surgical debridement was performed in all patients (see Fig. 1-b). When deemed necessary, the procedure was repeated until necrotic tissue had been removed and viable, healthy tissue obtained. All patients received broad-spectrum intravenous antibiotic therapy per institutional protocols for necrotizing soft tissue infections, and this therapy was subsequently adjusted based on culture results when available. White blood cell (WBC) and C-reactive protein (CRP) levels were evaluated from blood samples collected before surgery and on the 7th postoperative day. Patients not using VAC underwent daily debridement, and dressings were changed twice daily (Fig. 1-c).

Vacuum-assisted closure (VAC) therapy was initiated based on clinical judgment following adequate surgical debridement. In our clinical practice, VAC therapy was applied when the wound bed demonstrated viable, well-perfused tissue without evidence of ongoing necrosis, uncontrolled purulence, or active bleeding, and when satisfactory infection control was achieved. Patients were also required to be hemodynamically stable and suitable for regular wound assessment. In contrast, patients with severe systemic instability or rapidly progressive infection requiring frequent surgical reassessment were managed with conventional wound care. These clinical selection criteria may have influenced treatment allocation and should be considered when interpreting the results. The exact timing of VAC initiation and the total duration of therapy could not be consistently determined for all patients.

In this study, VAC therapy was delivered using a conventional negative-pressure wound therapy system without instillation or irrigation. The wound area to be treated with VAC is first surgically debrided, a process that involves removing necrotic tissue and exposing a layer of viable, well-perfused tissue. Before the procedure, haemostasis is achieved, and the wound is prepared by irrigation with antiseptic solutions. A sterile sponge composed of polyurethane or polyvinyl alcohol is meticulously shaped to conform to the contours of the wound cavity and subsequently positioned on the wound surface. The sponge is designed to ensure complete contact with the wound surface while avoiding contact with intact skin. The sponge is entirely covered with a transparent, adhesive polyurethane film, creating an airtight environment. This step is critical for the system to generate adequate negative pressure. A small incision is made in the film, through which a catheter is inserted. The catheter is connected to the pump unit, which generates negative pressure. The Exsudex vacuum pump (Haromed bvba, Gent, Belgium) typically applies intermittent negative pressure in the range of 80–120 mmHg. The device is set to a 10-minute cycle of negative pressure, followed by a 2-minute rest period. After system initiation, subatmospheric pressure is generated within the wound cavity. The vacuum dressing was changed every 72 h by a healthcare team that had undergone professional training in VAC, and the wound site was assessed for additional debridement during each change (see Fig. 1-d).

### Statistical analysis

The statistical analysis of the study was conducted using IBM Statistical Package for the Social Sciences (SPSS) version 25 software. Categorical variables were reported as frequencies and percentages, whereas continuous variables were expressed as mean ± standard deviation (SD) or as median values with their minimum–maximum ranges. The normality of the data distribution was evaluated using the Kolmogorov–Smirnov test. The Mann–Whitney U test was used to compare continuous variables that did not follow a normal distribution. In contrast, Student’s t-test was used for those that met the criteria for normality. The distribution of categorical variables between groups was compared using the chi-square test or Fisher’s exact chi-square test when the expected cell frequency was low. The paired t-test, also known as the Wilcoxon signed-rank test, was used to analyse changes between preoperative and postoperative values. In the statistical analyses, a p-value of < 0.05 was considered statistically significant.

## Results

In this study, 268 patients with a mean age of 62.5 ± 14.5 years were included, of whom 160 were assigned to Group 1 and 108 to Group 2. The mortality rate was 7.4% in Group 2 and 16.9% in Group 1, demonstrating a statistically significant difference between the groups (*p* = 0.038). The mean number of debridement procedures performed until discharge was 1.1 ± 0.8 in Group 1 and 1.8 ± 0.9 in Group 2 (*p* < 0.001). Additionally, the length of hospitalization was measured as 11.6 ± 9.4 days in Group 1 and 14.5 ± 9.6 days in Group 2. The rate of orchiectomy was significantly higher in Group 1 than in Group 2 (25.0% vs. 10.2%, *p* = 0.0017). No statistically significant differences were identified between the groups for sex, diabetes mellitus (DM), hypertension (HT), ICU admission, incidence of septic shock, or the number of colostomy procedures performed. The sociodemographic and clinical data for the groups are detailed in Table 1.

In a multivariate logistic regression analysis that included variables such as age, diabetes mellitus, hypertension, ICU admission, septic shock, FGSI score, and VAC therapy, it was found that only ICU admission (OR 6.40, 95% CI 1.52–26.89, *p* = 0.011) and FGSI score (OR 5.58, 95% CI 2.90–10.71, *p* < 0.001) were independently associated with mortality (Table 2). VAC therapy did not emerge as an independent predictor of mortality (OR 0.58, 95% CI 0.16–2.10, *p* = 0.409). The model demonstrated good explanatory power (Nagelkerke pseudo R² = 0.62).

**Fig. 2:**
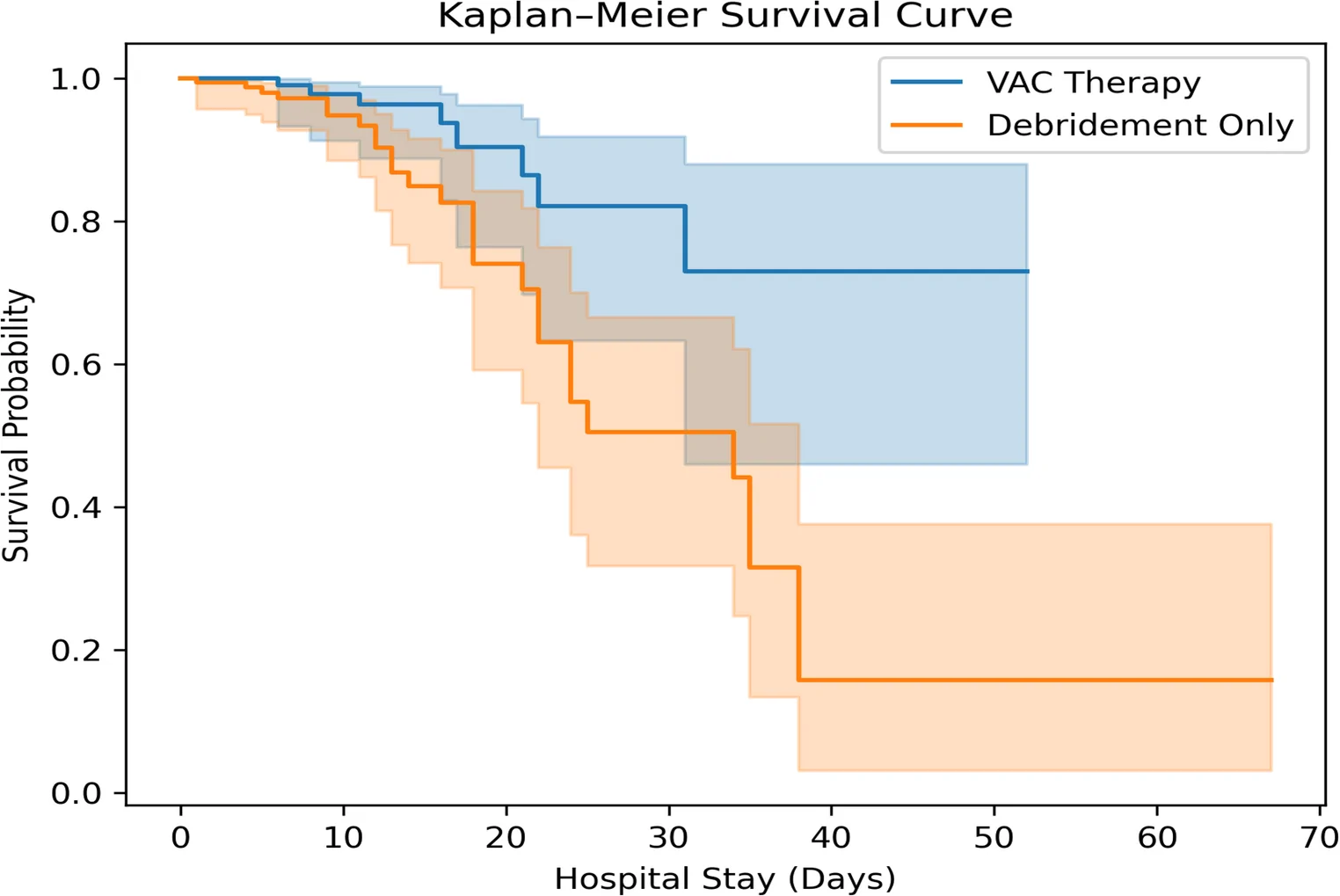
Kaplan–Meier survival curves comparing VAC therapy and debridement-only groups in patients with Fournier’s gangrene. The log-rank test demonstrated a statistically significant difference between groups (*p* = 0.002)

A Kaplan–Meier analysis using hospital length of stay as the time scale suggested a trend toward higher in-hospital survival in the VAC group; however, the curves substantially overlapped (Fig. 2). The difference between the groups was statistically significant according to the log-rank test (*p* = 0.002).

Following assessment of laboratory parameters, no significant differences were observed between groups in preoperative and postoperative C-reactive protein (CRP) levels, white blood cell (WBC) counts, or Fournier Gangrene Severity Index (FGSI) scores. In Group 1, the preoperative WBC count was 17437.5 ± 8779.4, while the postoperative WBC count was 14318.8 ± 7825.4.

**Table 1:** Sociodemographic and clinical findings of the groups

Data	Group 1 (*N* = 160)	Group 2 (*N* = 108)	*p*
Age (years, mean ± SD)	63.7 ± 14.2	60.7 ± 14.9	0.1006
Gender (Male, *N*, %)	107 (67.3%)	84 (77.8%)	0.0846
Diabetes mellitus (*N*, %)	126 (78.8%)	85 (78.7%)	0.9999
Hypertension (*N*, %)	89 (55.6%)	55 (50.9%)	0.5275
ICU admission (*N*, %)	63 (39.4%)	38 (35.2%)	0.5716
Septic shock (*N*, %)	36 (22.5%)	19 (17.6%)	0.4113
Mortality (*N*, %)	27 (16.9%)	8 (7.4%)	0.038
Number of debridements (mean ± SD)	1.1 ± 0.8	1.8 ± 0.9	< 0.001
Orchiectomy (*N*, %)	40 (25.0%)	11 (10.2%)	0.0017
Colostomy (*N*, %)	14 (8.8%)	8 (7.4%)	0.6437
Laboratory values (mean ± SD)			
Preoperative WBC (n)	17437.5 ± 8,779.4	17296.3 ± 9196	0.7274
Postoperative WBC (n)	14318.8 ± 7825.4	14324.1 ± 8265.3	0.8829
Preoperative CRP (mg/L)	131.5 ± 118.4	140.1 ± 118.4	0.3665
Postoperative CRP (mg/L)	111.5 ± 112	113.9 ± 107.3	0.5059
FGSI	6.4 ± 2	6.3 ± 1.3	0.459
Length of stay (days, mean ± SD)	11.6 ± 9.4	14.5 ± 9.6	0.0005

*Abbreviations*: *CRP* C-reactive protein, *FGSI* Fournier gangrene severity index, *SD* Standard deviation, *WBC* white blood cell

In the present study, the aetiology of FG was examined, with perianal abscess identified in 37.96% of cases, trauma in 17.8%, epididymo-orchitis in 9.6%, skin infections in 15.6%, hydrocele aspiration in 0.74%, and 0.4% developing after orchiectomy. A further 17.9% were evaluated as having an undetermined aetiology (idiopathic).

**Table 2:** Multivariate logistic regression analysis for predictors of mortality

Variable	Odds Ratio	95% CI	*p*-value
Age	1.02	0.97–1.08	0.420
Diabetes mellitus	3.25	0.40–26.11	0.267
Hypertension	0.33	0.09–1.26	0.106
ICU admission	6.40	1.52–26.89	**0.011**
Septic shock	0.77	0.19–3.05	0.709
FGSI score	5.58	2.91–10.71	**< 0.001**
VAC therapy	0.58	0.16–2.10	0.409

*Abbreviations*: *CI* Confidence interval, *FGSI* Fournier gangrene severity index, *ICU* Intensive care unit, *OR* Odds ratio, *VAC* Vacuum-assisted closure

Variables entered into the model: age, diabetes mellitus, hypertension, ICU admission, septic shock, FGSI score, and VAC therapy

**Fig. 3:**
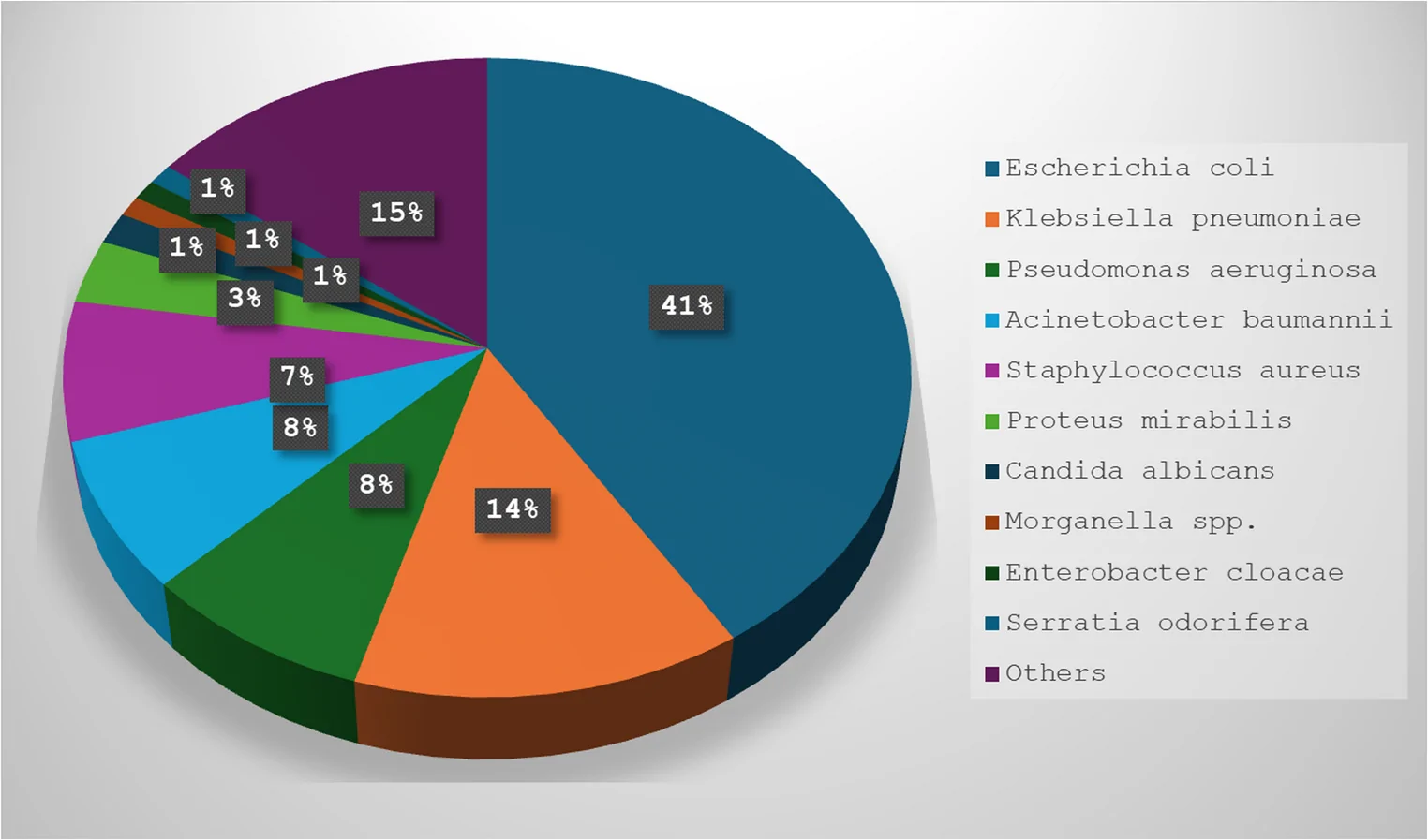
Distribution of isolated microorganisms

From a microbiological perspective, no causative agent was detected in up to 37.7% of patients. The most frequently isolated agents in the study were Escherichia coli in 75 (27.9%) patients, Klebsiella pneumoniae in 25 (9.3%) patients, Pseudomonas aeruginosa in 15 (5.5%) patients, and Acinetobacter baumannii in 14 (5.2%) patients. In contrast, Staphylococcus aureus, Proteus mirabilis, Candida albicans, and other single agents were reported at lower rates. Gram-negative bacilli were predominantly identified, while gram-positive bacteria and fungal agents were isolated less frequently. The distribution of isolated microorganisms is illustrated in Fig. 3.

## Discussion

Fournier’s gangrene is a rapidly progressing, life-threatening necrotising soft tissue infection affecting the perineal, genital, and perianal regions. It is associated with high mortality rates if not diagnosed early and treated with urgent surgical debridement. The underlying cause of the disease is considered to be polymicrobial, and it predominantly occurs in cases with predisposing factors such as diabetes mellitus, chronic renal failure, immunodeficiency, malignancy, or alcoholism [11]. A large-scale analysis based on US population data by Sorensen and Krieger reported that, despite modern technology, mortality rates associated with FG remain high today. They also reported that advanced age and comorbidities are negative prognostic factors [1].

As evident in the existing literature, there is considerable heterogeneity in the reported mortality rates for FG. A study conducted in the United States involving 650 patients reported a mortality rate of 10% [12]. A comprehensive review of 1,641 patients’ findings revealed a mortality rate of 7.5% associated with FG. In a German study involving 86 patients, the mortality rate was 16.3% [13], and in an Indonesian study involving 145 male patients, the mortality rate was 26.2% [14]. An analysis of 169 cases conducted in our country yielded a mortality rate of 11.8% [15], while a subsequent study of 193 cases revealed a mortality rate of 19.2% [16].

Previous studies from Turkey have reported outcomes of Fournier’s gangrene based on relatively small patient cohorts; for example, Yazici et al. reported clinical outcomes in only 10 patients [17]. In contrast, the present study includes a substantially larger cohort, which may allow for a more comprehensive evaluation of clinical outcomes and enhance the generalizability of the findings. This difference in sample size should be taken into account when interpreting comparisons with the existing literature.

In the present study, a total of 35 patients died due to FG, with 27 of these cases occurring in the debridement group and 8 in the VAC plus debridement group. The overall mortality rate related to FG was calculated to be 13.06%. Rapid diagnosis and successful emergency surgical debridement of the affected tissue are two critical factors that affect the treatment success of FG. A high mortality rate of 12–20% has been reported when early surgical intervention is not performed [18]. Furthermore, factors such as age, comorbidities, anatomical distribution, the development of sepsis, and fungal infection are among the other causes that increase mortality. Consequently, the mortality rate associated with FG identified in our study may be considered normal compared with rates in different regions of our country and worldwide.

In 1997, VAC was introduced into clinical practice for the treatment of acute and chronic wounds in an experimental pig model. The study aimed to reduce wound size by bringing the wound edges closer together, promote granulation tissue formation at the wound bed for skin grafting, increase microcirculation, reduce oedema, and remove infected tissue [6, 19, 20].

VAC therapy contributes to wound healing through several mechanisms, including the reduction of oedema via negative pressure, stimulation of granulation tissue formation, reduction of bacterial load, and control of wound exudate [5, 6]. Clinical studies have demonstrated that the utilisation of VAC leads to a substantial reduction in mortality rates among patients afflicted with necrotising fasciitis [18], yields superior outcomes in terms of wound healing in cases of tissue loss due to scalp trauma [21], and facilitates expedited healing in the context of diabetic foot treatment [22], while concomitantly accelerating the process of healing by augmenting granulation tissue in cases of osteonecrosis [23]. The present study demonstrated an association between vacuum-assisted closure (VAC) therapy and lower observed mortality among selected patients with Fournier’s gangrene. However, given the study’s retrospective design, a direct causal relationship between VAC therapy and mortality reduction cannot be established. The potential benefits of VAC therapy may include improved wound management, such as enhanced drainage, reduced local edema, and the promotion of granulation tissue, which may, in turn, indirectly contribute to better systemic infection control when applied under appropriate clinical conditions [24].

A significant limitation of this study is the potential for selection bias. Patients eligible for VAC therapy were more likely to be hemodynamically stable and suitable for repeated wound evaluation. In contrast, those with severe systemic instability or rapidly progressive sepsis may not have been candidates for VAC therapy and were therefore managed with conventional wound care. This inherent difference in baseline systemic condition may have contributed to the observed mortality differences between the groups and must be considered when interpreting the results.

Fournier’s gangrene is a predominantly polymicrobial infection with a significant anaerobic component [2]. In infected wounds, VAC therapy may theoretically worsen ongoing infection if adequate debridement and infection control are not achieved. In the present study, routine anaerobic cultures were not consistently available, and the decision to initiate VAC therapy was based on careful clinical assessment of the wound after repeated debridement. This represents a significant limitation and underscores the need for meticulous patient selection and close monitoring when VAC therapy is considered in the management of Fournier’s gangrene.

However, the present study reveals that the VAC group demonstrates a higher frequency of debridements and a prolonged hospital stay. Although certain studies indicate that VAC therapy may offer survival advantages, its direct effect on mortality remains uncertain, especially when accounting for clinical severity [25]. Although VAC/VSD methods accelerate granulation tissue formation in the wound bed, frequent surgical intervention may be necessary to maintain dressing stability and prevent contamination. A case report and systematic review by Altomare et al. also indicated that while VAC offers advantages in wound care, it may increase costs and prolong care duration [26]. It is imperative to consider the disadvantages of the treatment, which may result in a protracted treatment process, alongside its acknowledged advantages in wound management. The observed difference in orchiectomy rates between the groups is more likely attributable to the extent of local disease at presentation than to the effect of VAC therapy itself. Orchiectomy was performed based on intraoperative findings of testicular involvement and tissue viability, independent of the wound management strategy.

The most frequently isolated microorganism in the present study was Escherichia coli, followed by Klebsiella pneumoniae and Pseudomonas aeruginosa. This distribution is consistent with the results reported in recent studies. Hong et al. demonstrated the predominance of Gram-negative bacilli and, in particular, the role of multidrug-resistant (MDR) strains in increasing mortality [11]. Eke’s extensive review, published in 2000, also reported E. coli as the most common causative agent. However, studies over the last decade have emphasised the increase in MDR bacteria [2, 11]. The predominance of Gram-negative bacteria was also demonstrated, thereby supporting the necessity for broad-spectrum agents in empirical treatment.

In the present study, FGSI scores between the two groups were comparable, and no demonstrable direct effect on mortality was observed. However, a systematic review by Franco-Buenaventura et al. emphasised that FGSI is a reliable predictor of mortality [9]. In a similar vein, Schneidewind et al. observed in their study examining FG treatment approaches in Europe that FGSI and comorbidities were significant predictors of patient prognosis [27]. Furthermore, Czymek et al. suggested that female gender may be a risk factor. However, more recent publications have stated that this finding cannot be generalised [4]. The lack of marked differences observed in our study may be attributable to the study’s retrospective design and the relatively small sample size.

Although the VAC group exhibited reduced mortality in unadjusted comparisons, multivariate analysis demonstrated that VAC therapy was not an independent predictor of survival. This discrepancy likely reflects differences in baseline clinical status and patient selection. Patients allocated to the VAC group were more likely to be hemodynamically stable and to have better-controlled local infections, which may have contributed to improved unadjusted outcomes. In contrast, disease severity—as quantified by the FGSI score—and the requirement for ICU admission emerged as the primary independent determinants of mortality. These findings suggest that the apparent survival benefit associated with VAC therapy may be influenced by underlying clinical characteristics rather than representing a direct causal effect.

In this context, high-risk patients with Fournier’s gangrene may be defined as those with advanced age, multiple comorbidities such as diabetes mellitus or chronic renal failure, extensive local disease requiring repeated debridement, or prolonged hospitalization [11]. In these selected patients, VAC therapy may offer advantages in wound management; however, its use should be individualized and carefully weighed against potential risks, treatment duration, and cost.

This study has several significant limitations. First, its retrospective design limits the ability to establish causal relationships between VAC therapy and clinical outcomes. A detailed, standardized assessment of wound severity, including wound size, depth, extent of tissue involvement, and local anaerobic infection status before VAC initiation, could not be uniformly performed. Second, routine anaerobic cultures were not consistently available, and the decision to initiate VAC therapy was based primarily on clinical wound assessment after repeated debridement. Third, a significant selection bias is likely, as patients who were hemodynamically stable and suitable for repeated wound evaluation were more likely to receive VAC therapy. In contrast, critically unstable patients may not have been candidates for this intervention. These limitations should be considered when interpreting the observed differences in outcomes between the groups.

Despite these limitations, the relatively large sample size of this study, compared to many previously published series, may enhance the generalizability of the findings.

## Conclusion

In conclusion, while VAC therapy was associated with reduced observed mortality in unadjusted analyses, it was not identified as an independent predictor of mortality after adjusting for clinical severity. The FGSI score and the requirement for ICU admission emerged as the most significant determinants of mortality within this cohort. Although VAC therapy may enhance wound management, its effect on survival appears to be influenced by the baseline severity of the disease. Prospective, multicenter studies are necessary to more precisely delineate the role of VAC therapy in the management of Fournier’s gangrene.

## Supplementary Information

Supplementary Material 1.

Supplementary Material 2.
