# Supplementary material for: Outcomes of vacuum-assisted closure therapy (VAC) in patients with Fournier’s gangrene: a retrospective cohort study
Source: Ir J Med Sci. 2026 Apr 27;195(4):2497–504. doi: 10.1007/s11845-026-04416-w (PMC13588902; doi:10.1007/s11845-026-04416-w)
Supplement: Supplementary file 1 [file 11845_2026_4416_MOESM2_ESM.pdf]

Authorship Form

Irish Journal of Medical Science

Springer

Manuscript ID Number: IJMS-D-26-00850 ....

Article Title: (first few words) Outcomes of Vacuum-Assisted Closure Therapy (VAC) in Patients with Fournier's Gangrene

First Author: Emrah Küçük ....

E-mail: emrah.kucuk@samsun.edu.tr ....

AUTHORSHIP

I, the undersigned author(s), certify that:

- **I have seen and approved the final version of the manuscript, and all subsequent versions;**
- **I have made substantial contributions to conception and design, or acquisition of data, or analysis and interpretation of data;**
- **I have drafted the article or revised it critically for important intellectual content.**
- **I agree to be accountable for all aspects of the work in ensuring that questions related to the accuracy or integrity of any part of the work are appropriately investigated and resolved.**

I accept public responsibility for it, and believe it represents valid work. As an author of this article, I certify that none of the material in the manuscript has been previously published, nor is included in any other manuscript. I certify that this manuscript is not under consideration for publication elsewhere, nor has it been submitted or accepted in another publication in any form. The rights or interest in the manuscript have not been assigned to any third party.

Moreover, should the editor of *Irish Journal of Medical Science* request the data upon which the manuscript is based, I shall produce it. I also certify that I have read and complied with the copyright information, as found on the journal home page website.

After submission of this agreement signed by all authors, changes of authorship or in the order of the authors listed will not be accepted by Springer.

☑ [signature]
Author's signature
Emrah Küçük 02/28/2026
Printed name & date

☑ furkan
Author's signature
Ahmet Furkan Akıllı 02/28/2026
Printed name & date

☑ [signature]
Author's signature
Reha Ordulu 02/28/2026
Printed name & date

☑ mesut
Author's signature
Mesut Şengül 02/28/2026
Printed name & date

☑ [signature]
Author's signature
Muhammed Esad Kayhan 02/28/2026
Printed name & date

☑ [signature]
Author's signature
Ekrem Akdeniz 02/28/2026
Printed name & date

☑ [signature]
Author's signature
Mahmut Ulubay 02/28/2026
Printed name & date

☑ [signature]
Author's signature
Mustafa Kemal ATilla 02/28/2026
Printed name & date

Completed forms can be scanned and included as a pdf file during the online submission process as a supplemental file not for review, or submitted by fax to the editorial office: +91 44 42197763
